# Construction of T7-Like Expression System in *Pseudomonas putida* KT2440 to Enhance the Heterologous Expression Level

**DOI:** 10.3389/fchem.2021.664967

**Published:** 2021-07-16

**Authors:** Tianxin Liang, Jun Sun, Shuyun Ju, Shenyi Su, Lirong Yang, Jianping Wu

**Affiliations:** ^1^ Institute of Bioengineering, College of Chemical and Biological Engineering, Zhejiang University, Hangzhou, China; ^2^ Hangzhou Global Scientific and Technological Innovation Center, Zhejiang University, Hangzhou, China; ^3^ Hwa Chong Institution, Singapore, Singapore

**Keywords:** *Pseudomonas putida* KT2440, chassis, heterologous expression, synthetic biology, RNA polymerase, difficult-to-express protein

## Abstract

*Pseudomonas putida* KT2440 has become an attractive chassis for heterologous expression with the development of effective genetic manipulation tools. Improving the level of transcriptional regulation is particularly important for extending the potential of *P. putida* KT2440 in heterologous expression. Although many strategies have been applied to enhance the heterologous expression level in *P. putida* KT2440, it was still at a relatively low level. Herein we constructed a T7-like expression system in *P. putida* KT2440, mimicking the pET expression system in *Escherichia coli*, which consisted of T7-like RNA polymerase (MmP1) integrated strain and the corresponding expression vector for the heterologous expression enhancement. With the optimization of the insertion site and the copy number of RNA polymerase (RNAP), the relative fluorescence intensity (RFI) of the super-folder green fluorescent protein (sfGFP) was improved by 1.4-fold in MmP1 RNAP integrated strain. The induction point and IPTG concentration were also optimized. This strategy was extended to the gene-reduced strain EM42 and the expression of sfGFP was improved by 2.1-fold. The optimal RNAP integration site was also used for introducing T7 RNAP in *P. putida* KT2440 and the expression level was enhanced, indicating the generality of the integration site for the T7 expression system. Compared to other inducible expression systems in KT2440, the heterologous expression level of the Mmp1 system and T7 system were more than 2.5 times higher. Furthermore, the 3.6-fold enhanced expression level of a difficult-to-express nicotinate dehydrogenase from *Comamonas testosteroni* JA1 verified the efficiency of the T7-like expression system in *P. putida* KT2440. Taken together, we constructed and optimized the T7-like and T7 expression system in *P. putida*, thus providing a set of applicable chassis and corresponding plasmids to improve recombinant expression level, expecting to be used for difficult-to-express proteins.

## Introduction

*Pseudomonas putida* KT2440, a non-pathogenic soil bacteria, is an attractive chassis for heterologous expression (Timmis, 2002; Dammeyer et al., 2011; Poblete-Castro et al., 2012; Lieder et al., 2015) due to its rapid growth (Martins Dos Santos et al., 2004), metabolic diversity (Poblete-Castro et al., 2012; Nikel et al., 2014, Nikel et al., 2016), rapid generation ability of nicotinamide adenine dinucleotide (Ebert et al., 2011), the operability of genetic manipulation (Martínez-García and de Lorenzo, 2019) and robustness to extreme environments (Nikel and de Lorenzo, 2018).

Many efforts have been made to enhance the heterologous expression level in *P. putida* KT2440 by regulating the transcriptional level or translation level. To improve the integration expression, the constitutive promoter libraries have been constructed (Zobel et al., 2015) and the T7 RNA polymerase has been integrated into the genome of *P. putida* KT2440 (Herrero et al., 1993; Christina et al., 2012). Additionally, to enhance the plasmid expression, suites of broad-host-expression plasmids (Calero et al., 2016; Cook et al., 2018), a library of synthetic promoters (Elmore et al., 2017), and the dual-inducible duet-expression system have been generated (Gauttam et al., 2020). The modification of the ribosome-binding site (Calero et al., 2016) and codon optimization (Dammeyer et al., 2011) were also carried out to enhance the expression at the translational level. However, the expression level of *P. putida* KT2440 is still low, limiting the application of *P. putida* as a chassis for heterologous expression in industry.

T7 RNA polymerase expression system is an important and high-efficient expression system in model strains such as *Escherichia coli* (Wang et al., 2018; Shilling et al., 2020). However, the T7 RNA polymerase-mediated expression system is not always efficient for some non-model microbial strains (Zhao et al., 2017). For example, the T7 expression system constructed in *P. putida* KT2440 was low efficiency and was rarely used (Christina et al., 2012). Recently, broad-host T7-like systems including the MmP1 expression system have been developed in *Halomonas* sp. TD01 and *P. entomophila* LAC31 (Zhao et al., 2017). Furthermore, the MmP1 expression system constructed in the plasmid has successfully contributed to the enhancement of heterologous expression of nicotinate dehydrogenase (NDHase) in *C. testosteroni* CNB-2 (Lu et al., 2020). Given the high efficiency and versatility, it is supposed that the T7-like system might be suitable for the heterologous expression in *P. putida* KT2440.

In this study, we attempted to construct the T7-like expression system and evaluate the heterologous expression level in *P. putida* KT2440. MmP1 RNAP cassette was successfully integrated into the genome of *P. putida* KT2440, which significantly improved the expression level of super-folder green fluorescent protein (sfGFP). The insertion site and the copy number of MmP1 RNAP cassette were then optimized to improve the efficiency of the MmP1 expression system. We successfully obtained the T7-like expression system in *P. putida* KT2440, which consisted of the MmP1 RNAP integrated strain and corresponding plasmid. We also utilized the strategy to obtain a T7 expression system in *P. putida* KT2440 with T7 RNAP inserted in the optimal site. These expression systems were much more efficient than those previously reported in *P. putida* KT2440. The Mmp1 expression system was also constructed in the *P. putida* EM42. The expression level of difficult-to-express NDHase was successfully enhanced by using the MmP1 expression system in both *P. putida* KT2440 and *P. putida* EM42, demonstrating the high efficiency of the expression system.

## Materials and Methods

### Strains, Culture Conditions, and Chemicals

*E. coli* strain DH5α was used for molecular cloning and plasmid propagation throughout this study. *P. putida* KT2440 and EM42 were used for the characterization of T7 and MmP1 RNAP-promoter pairs. Luria-Bertani (LB) medium was used for culturing *E. coli* and *P. putida*. Antibiotics were supplemented at the following concentrations when needed: for *E. coli*, 50 μg/ml kanamycin, 15 μg/ml tetracycline; for *P. putida*, 100 μg/ml kanamycin, 25 μg/ml tetracycline, 100 μg/ml spectinomycin, 50 μg/ml gentamicin. The inducer for P_trc_ promoter was isopropyl β-D-1-thiogalactopyranoside (IPTG) and 3-methylbenzoate was for Xyls/*P*_*m*_, arabinose was for AraC/*P*_*araB*_, and rhamnose was for RhaS/*P*_*rha*_. *E. coli* was grown at 37°C, and *P. putida* KT2440 and EM42 were incubated at 30°C. The DNA polymerase PrimeSTAR^®^ Max was purchased from Takara Bio Inc (Dalian, China). The Green Taq Mix and the One Step Cloning Kit were purchased from Vazyme Biotechnology (Nanjing, China). The Fast Digest *Dpn*I and the PageRuler™ Prestained Protein Ladder 10–180 kDa were purchased from Thermo Scientific (United States).

### Construction of Plasmids

All the strains and plasmids used in this study are listed in Supplementary Table 1. All the integration strains are listed in Supplementary Table 1. All the primers used to construct vectors are listed in Supplementary Table 2. Plasmid pSEVA-gRNAF was used as the backbone for plasmid construction. Plasmid pSEVA64-vdh was derived from pSEVA-gRNAF by replacing the original N20 sequence with “GTG​GAA​CGC​CCC​GGT​GAT​AC” *via* inverse PCR using primers vdh-gF/vdh-gR. The 800 bp length upstream and downstream homologous arms of *vdh* acquired from the Pseudomonas Genome Database were amplified using primers vdh-1F/vdh-ZR, vdh-ZF/vdh-2R, and connected *via* overlap-extension (SOE) PCR. The N20 sequence was designed by CasOT (Xiao et al., 2014). A similar method was applied to construct the plasmids pSEVA64-5003 and pSEVA64-5007. T7-like RNAP cassette and promoter were cloned from the plasmid pMmP1-p321, which were donated by the Chen team (Zhao et al., 2017). The RNAP module of T7 and MmP1 was inserted between the upstream homologous arm and the downstream homologous arm to generate the gene inserting vector pSEVA64-T7-vdh, pSEVA64-MmP1-vdh, pSEVA64-MmP1-5003, pSEVA64-T7-5003, and pSEVA64-Mmp1-5007. The verification plasmids containing RNAP modules and their promoter modules (the reporter protein is the sfGFP) were assembled on the plasmid backbone pSEVA64 (Martínez-Garćía et al., 2015) using One Step Cloning Kit to create the plasmids pSEVA64-DE-pRO, pSEVA64-MmP1-pRO. Removed the RNAP module of pSEVA64-DE-pRO, pSEVA64-MmP1-pRO to create the plasmids pSEVA64-DE-sfGFP and pSEVA64-MmP1-sfGFP. Similarly, the other five inducible expression systems (LacI^q^-*P*_*trc*_, LacI^q^-*P*_*trc*_, Xyls/*P*_*m*_, AraC/*P*_*araB*_, RhaS/*P*_*rha*_) were assembled on the upstream of sfGFP with the plasmid backbone pSEVA64 to create the plasmid pSEVA644-sfGFP, pSEVA644R-sfGFP, pSEVA648-sfGFP, pSEVA6410-sfGFP, and pSEVA64-Rha-sfGFP.

### Construction of Platform Strains

The platform strains including KTVT and KTVM were constructed by integrating the RNAP module of T7 and MmP1 into the *vdh* site of the genome of *P. putida* KT2440, respectively. The integration was carried out by CRISPR/Cas9 system (Sun et al., 2018). The integrated strain KTCM, KTFM, EMCM, EMFM were constructed by integrating the RNAP module of MmP1 into the PP_5003 and the PP_5007 site of *P. putida* KT2440 and EM42, respectively. The other four *P. putida* KT2440 mutants, KTCVM, KTVFM, KTCFM, KTCFVM were constructed by the different combinations of the integration sites. The integrated strain KTCT was generated by introducing the T7 RNAP cassette into the PP_5003 site of *P. putida* KT2440.

### Coarse Characterization

**FIGURE 1:**
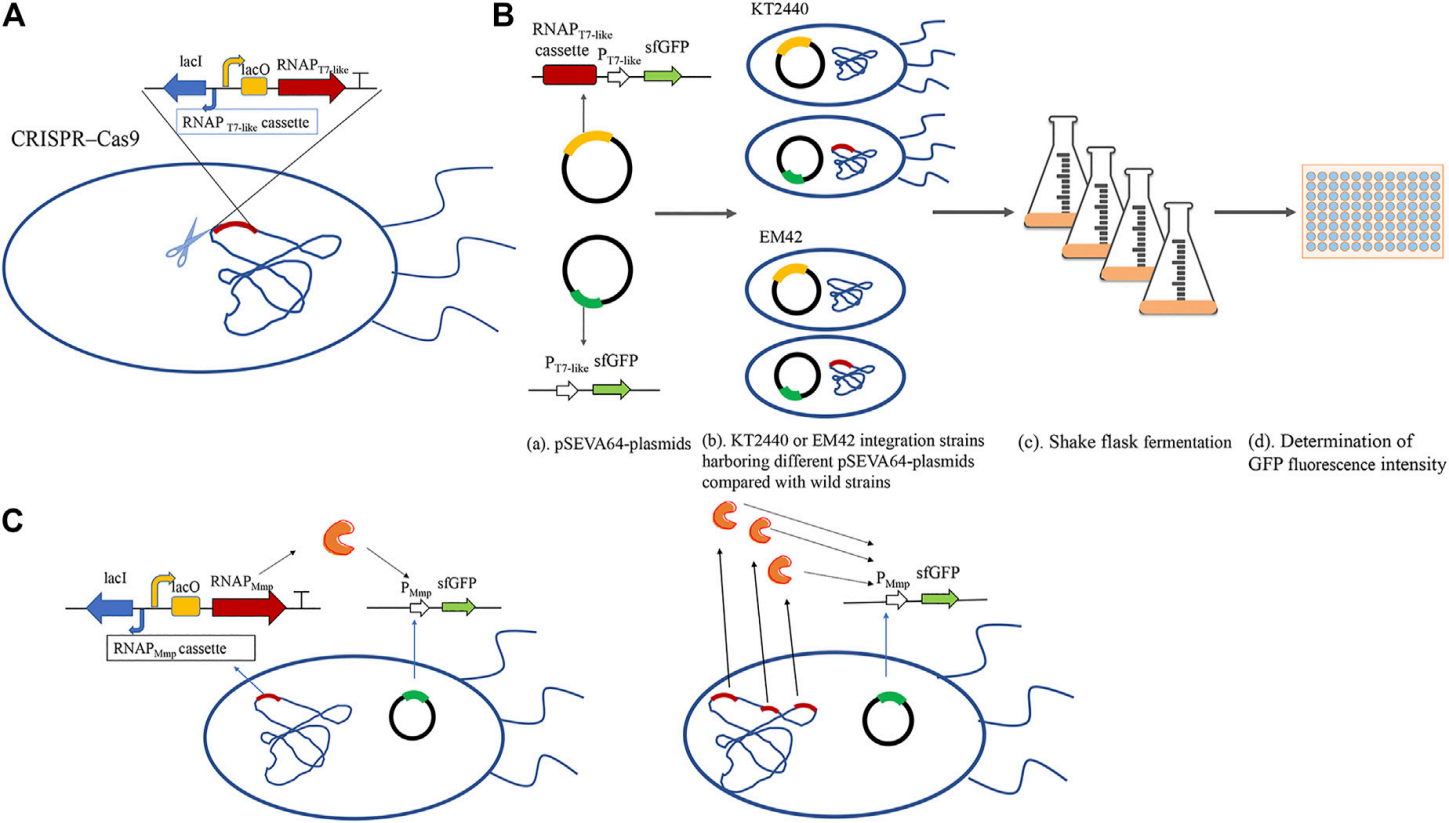
The schematic diagram of the MmP1 expression system constructed in *P. putida*. **(A)** Construction of integrated strain by integrating the RNAP cassette into nonessential gene of the genome in *P. putida* by CRISPR/Cas9; **(B)** Flowchart about the flask shake fermentation of sfGFP with different RNA polymerase cassette; (a) Construction of verified plasmids: the upper contains both RNAP cassette and promoter modules; the lower contains only promoter modules; (b) The verified plasmids were into wild-type strains and RNAP integrated strains, respectively; (c) Shake flask experiments were carried out by inoculating with 2% of the seed cultures at 30°C; (d) Cells were harvested and diluted two times. 200 μl of the diluted liquid was added into the 96-well microtiter plates and measured the absolute fluorescence intensity (AFI) and cell density (OD_600_) by microplate reader using the excitation wavelength at 488 nm and fluorescence emission at 510 nm. Finally, the relative fluorescence intensity (RFI) was calculated by the AFI/OD_600_.; **(C)**Construction of different copy numbers of MmP1 RNAP integrated in the genome of *P. putida.*

Plasmids pSEVA64-MmP1-pRO and pSEVA64-MmP1-sfGFP were transformed into *P. putida* KT2440, KTVM, respectively, using the electroporation method (Figure 1). Plasmids pSEVA64-DE-pRO and pSEVA64-DE-sfGFP were transformed into *P. putida* KT2440, KTVT, respectively. The candidate strains were screened in selection plates and identified by colony PCR. These strains were incubated in LB medium at 30°C, and 1 mM IPTG was added for the induction expression when the optical density reached OD_600_ = 0.4 (about 3 h). After 24 h of incubation, cells were harvested by centrifugation at 4°C and 12,000 × g for 10 min. The cell pellets were washed twice with phosphate buffer (pH 7.4) and then diluted two times. 200 μ of the diluted liquid was added into the 96-well microtiter plates and measured the absolute fluorescence intensity (AFI) and cell density (OD_600_) by a microplate reader using the excitation wavelength at 488 nm and fluorescence emission at 510 nm. Finally, the relative fluorescence intensity (RFI) was calculated by the AFI/OD_600_.

### Optimization of Induction Condition of Different Inducible Expression Systems in *P. putida* KT2440

For the Mmp1 expression system, the RNAP-integrated strains harboring the corresponding verification plasmids were grown in 5 ml LB with 50 μg/ml gentamicin overnight at 30°C. Then, shake flask experiments were carried out by inoculating 2% of the seed cultures at 30°C. 1 mM IPTG was added in the shake flasks at 0, 2, 4, 6, and 8 h after the inoculation at 30°C, respectively. The AFI and OD_600_ of the cultures were measured after 24 h of inoculation to obtain the optimal induction point. Next, the optimal IPTG concentration was obtained using a similar method. Different IPTG concentrations (1, 10, 25, 75, 100, 250, 500, 750, 1,000, 1,500, 2,000, 2,500, and 3,000 μM) were added at 4 h (the optimal induction point) after inoculating in shake flasks at 30°C. Then the optimal IPTG concentration was obtained by comparing the RFI of sfGFP.

For other inducible expression systems, *P. putida* KT2440 strains containing sfGFP expression plasmids (pSEVA644-sfGFP, pSEVA644R-sfGFP, pSEVA648-sfGFP, pSEVA6410-sfGFP, pSEVA64-Rha-sfGFP) were grown overnight in 5 ml LB with 50 μg/ml gentamicin. 2% of the seed cultures were inoculated into shake flask. Varying amounts of inducer were added 4 h after inoculating: IPTG (0, 250, 500, 750, 1,000, 1,500 μM) for pSEVA644-sfGFP, pSEVA644R-sfGFP, 3-methylbenzoate (0, 0.25, 0.5, 0.75, 1 mM) for pSEVA648-sfGFP, arabinose (0, 10, 50, 80, 100, 120 mM) for pSEVA6410-sfGFP, rhamnose (0, 3, 5, 10, 15 mM) for pSEVA64-Rha-sfGFP. Optimal inducer concentrations were obtained by comparing the RFI of sfGFP.

### The NDHase Activity Assay

The engineered strains (KTCM and EMCM), as well as the wild-type strains (KT2440 and EM42) harboring pSEVA64-JA-sfGFP plasmid, were cultured in 5 ml LB overnight at 30°C. 1 ml of the seed cultures were used to inoculate in 250 ml shake flasks. Then 0.5 mM IPTG was added at 4 h after inoculation for the induction expression at 18°C for 20 h. After the shake flask fermentation, 4 ml of the samples were harvested by centrifugation at 4°C and 4,000 rpm for 10 min. The cell pellets were resuspended with 1 ml of 0.1 M 3-cyanpyridine. To measure NDHase activity, 0.05 mM PMS was added into 1 ml resuspension with magnetic stirring for 2 h at 30°C. Cells were heated at 99°C for 5 min to terminate the reaction. High-performance liquid chromatography (HPLC) with a Pntulips QS-C18 column (4.6 mm × 250 mm, 5 μm) was used to detect the production of 6-hydroxy-3-cyanopyridine (6-HCP). Detection was performed using a wavelength of 260 nm with 1 ml/min of the liquid phase containing acetonitrile and water (10:90 v/v). One unit (U) of NDHase activity was defined as the amount of enzyme required to produce 1 μmol of 6-HCP in 1 min under the test conditions.

## Result

### Construction of MmP1 Expression System in *P. putida* KT2440

To study the effect of T7-like expression system containing T7-like RNAP cassette and T7-like promoter module in *P. putida* KT2440, the Mmp1 RNAP cassette was inserted either in the genome or plasmid. The Mmp1 RNAP cassette was first integrated into the *vdh* site (locus tag is PP_3357) in the genome by CRISPR/Cas9, resulting in the integrated strain KTVM (Figure 1A and Supplementary Figure 1). The corresponding plasmid containing P_MmP1_ promoter and *sfgfp* was transformed into the integrated strain KTVM to examine the effect of constructed system. The RNAP cassette coupled with *sfgfp* was also inserted in the plasmid pSEVA64, which was then transformed into *P. putida* KT2440.

**FIGURE 2:**
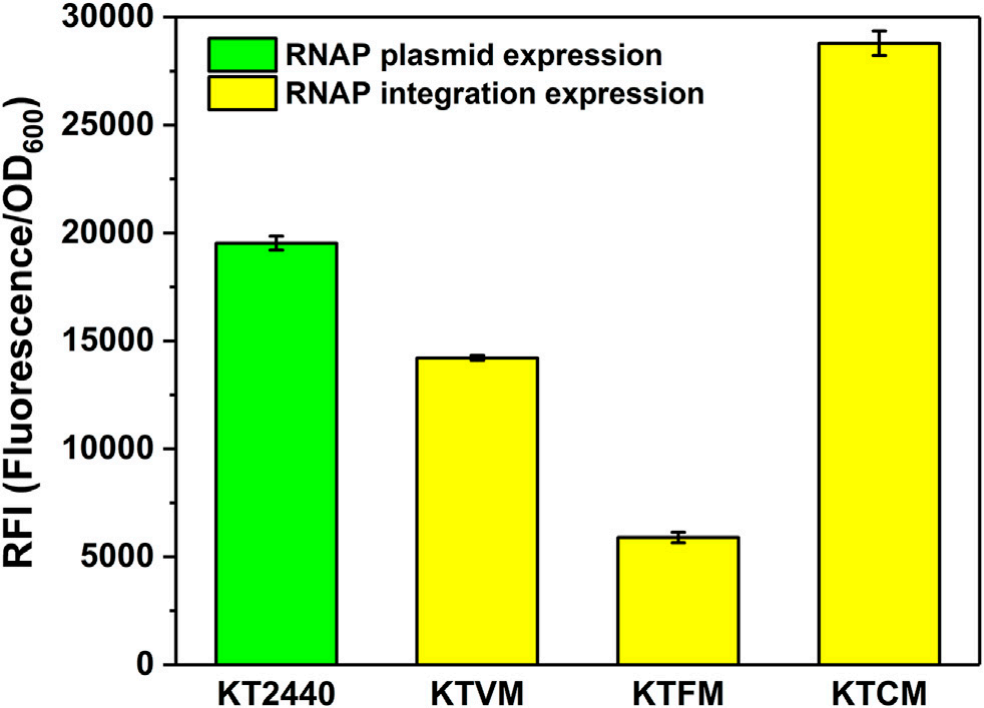
Optimization of the MmP1 RNAP casette integrated into *P. putida* KT2440 with different integration sites. KT2440: KT2440 harboring the plasmid containing the MmP1 RNAP module and sfGFP with MmP1 promoter; KTVM: KTVM with MmP1 RNAP cassette integrated into *vdh* site of KT2440 harboring the plasmid containing sfGFP with MmP1 promoter; KTFM: KTFM with MmP1 RNAP cassette integrated into *phaC1* site of KT2440 harboring the plasmid containing sfGFP with MmP1 promoter; KTCM: KTCM with MmP1 RNAP cassette integrated into *phaF* site of KT2440 harboring the plasmid containing sfGFP with MmP1 promoter.

The absolute fluorescence intensity (AFI) and cell density (OD_600_) were determined after shake-flask fermentation (Figure 1B). When the MmP1 RNAP cassette was expressed in the plasmid, the RFI value was slightly higher than that inserted into the genome (Figure 2). However, the large-size expression plasmid might decrease the transformation frequency (Walker and Klaenhammer, 2002), affect the quantity of the plasmid DNA isolated from the host strain (Yang and Yang, 2012), and was difficult to construct than smaller size plasmids. Although RNAP cassette inserted in the plasmid provided relatively higher sfGFP expression, considering the RNAP integrated into the genome was genetically stable and convenient to manipulate as the development of the genome editing technology. Hence, the MmP1 RNAP cassette integrated into the genome of *P. putida* KT2440 was chosen for further enhancing the heterologous expression.

### Optimization of the Mmp1 Expression System in *P. putida* KT2440

#### Optimization of the Insertion Site of MmP1 RNAP Cassette

The insertion sites were optimized to further improve the MmP1 expression system in *P. putida* KT2440 (Supplementary Table 3). Three nonessential genes including *phaC1*, *phaF*, *vdh* were selected as the insertion sites, which have been used to integrate other heterologous genes (Gong et al., 2016). The modified strains were named KTCM, KTFM, and KTVM with RNAP cassette inserted in *phaC1, phaF*, and *vdh,* respectively. Interestingly, the expression of sfGFP was improved 2-fold in KTCM relative to that in KTVM. Compared to the plasmid expression style, the expression of the sfGFP in KTCM was increased by 1.4-fold (Figure 2), whereas that in KTFM was decreased by 3.3-fold.

#### Optimization of the Copy Number of the MmP1 RNAP Cassette

**FIGURE 3:**
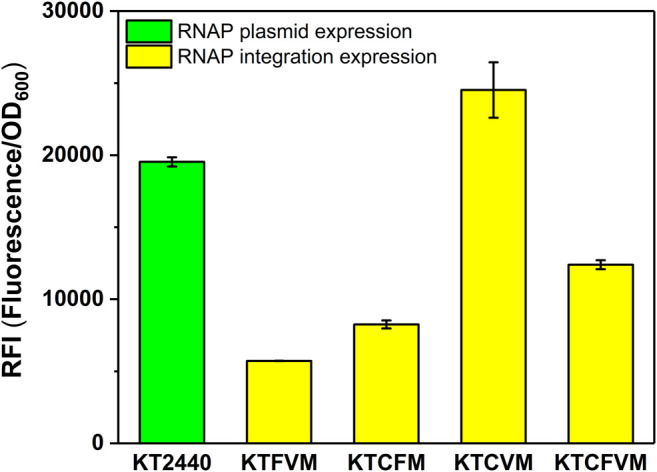
The effect of different copy numbers of Mmp1 RNAP on expression. KT2440: KT2440 harboring the plasmid containing the MmP1 RNAP module and sfGFP with MmP1 promoter; KTFVM: KTFVM with insertion sites of *phaF* and *vdh* harboring the plasmid containing sfGFP with MmP1 promoter; KTCFM: KTCFM with insertion sites of *phaC1* and *phaF* harboring the plasmid containing sfGFP with MmP1 promoter; KTCVM: KTCVM with insertion sites of *phaC1* and *vdh* harboring the plasmid containing sfGFP with MmP1 promoter; KTCFVM: KTCFVM with insertion sites of *phaC1*, *phaF* and *vdh* harboring the plasmid containing sfGFP with MmP1 promoter.

We also investigated the effect of the copy number of MmP1 RNAP cassette integrated into *P. putida* KT2440 on expression. Two copies of RNAP integrated strains were constructed including KTCFM with insertion sites of *phaC1* and *phaF*, KTCVM with insertion sites of *phaC1* and *vdh*, KTFVM with insertion sites of *phaF* and *vdh*, and three copies of RNAP integration strain, KTCFVM was constructed with the insertion sites of *phaC1, phaF,* and *vdh* (Figure 1C). Among them, the two-copy strain KTCVM displayed the highest expression level of sfGFP, 1.3-fold higher than the RNAP plasmid expression system (Figure 3). However, it was still slightly lower than the KTCM (Figure 2). Thus, the strain KTCM was selected for further investigation, and the MmP1 expression system in *P. putida* KT2440 consisting of the host strain KTCM and the plasmid pSEVA64-MmP1-sfGFP carrying P_MmP1_ promoter corresponding to MmP1 RNAP.

### Construction of the MmP1 Expression System in *P. putida* EM42

**FIGURE 4:**
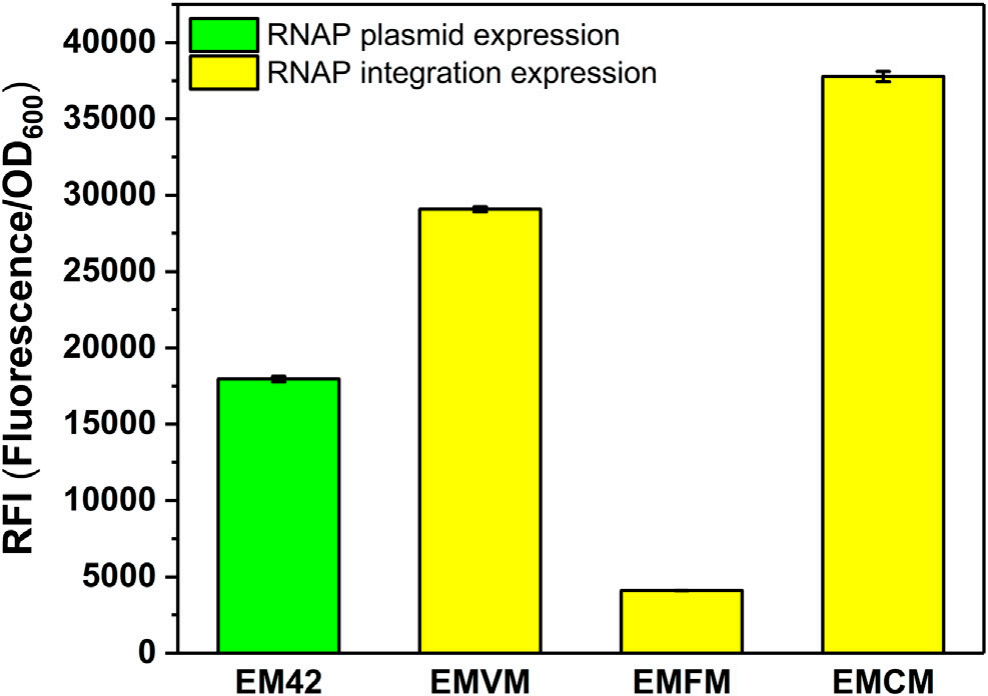
RFI of the MmP1 RNAP cassette integrated into *P. putida* EM42 with different integration sites. EM42: EM42 harboring the plasmid containing the MmP1 RNAP module and sfGFP with MmP1 promoter; EMVM: EMVM with insertion sites of *vdh* harboring the plasmid containing sfGFP with MmP1 promoter; EMFM: EMFM with insertion sites of *phaF* harboring the plasmid containing sfGFP with MmP1 promoter; EMCM: EMCM with insertion sites of *phaC1* harboring the plasmid containing sfGFP with MmP1 promoter.

To verify the versatility of the MmP1 expression system, we extended the MmP1 expression system to the gene-reduced strain EM42. The optimal RNAP integration site (*phaC1*) was utilized in EM42 resulting in the integrated strain EMCM. As a result, the RFI in EMCM was about 2.1-fold higher than that of the MmP1 RNAP plasmid expression in EM42 (Figure 4).

### Verification of the Commonality of the Strategy to Improve Expression Level With T7 RNAP

**FIGURE 5:**
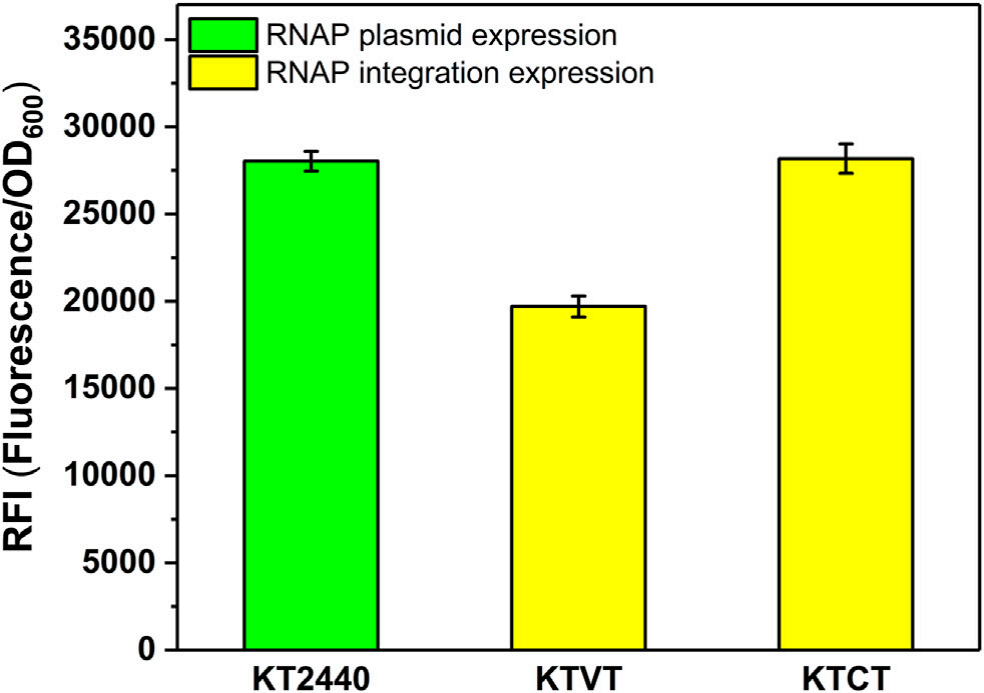
RFI of the T7 RNAP cassette integrated into *P. putida* KT2440 with different integration sites. KT2440: KT2440 harboring the plasmid containing the T7 RNAP module and sfGFP with T7 promoter; KTVT: KTVT with T7 RNAP cassette integrated into *vdh* site of KT2440 harboring the plasmid containing sfGFP with T7 promoter; KTCT: KTCT with T7 RNAP cassette integrated into *phaC1* site of KT2440 harboring the plasmid containing sfGFP with T7 promoter.

Similar to the construction of KTVM, KTCM, we introduced the T7 RNAP cassette into the genome of KT2440 to obtain KTVT and KTCT. To test whether the strategy of increasing heterologous expression level applied to T7 RNAP, the corresponding plasmid containing sfGFP was transformed into the integrated strains KTVT and KTCT. It turns out that the optimal insertion site was also effective for T7 RNAP. As shown in Figure 5, the RFI in KTCT was 1.4-fold higher than that in KTVT.

### Comparing the Optimized MmP1 Expression System and the T7 Expression System With Other Expression Systems in *P. putida* KT2440

The optimized MmP1 and T7 expression systems were compared with other inducible expression systems in *P. putida* KT2440 to understand its effect. The induction conditions were first optimized. The optimization of the induction condition of KT2440 was performed under different induction points (0–8 h) by using the MmP1 expression system. As shown in Supplementary Figure 4A, when IPTG was added at the induction point 4 h after inoculation, the final RFI value was highest, which was determined to be the optimal induced point (Supplementary Figure 4A).

Different inducer concentrations were tested for the effect on expression level and the optimal inducer concentration for six inducible expression systems in *P. putida* was 0.5 mM IPTG for MmP1 expression system, 1 mM IPTG for LacI^q^/*ptac* expression system, 2 mM IPTG for LacI^q^/*ptrc* expression system, 80 mM arabinose for AraC/*P*_*araB*_ expression system, 5 mM rhamnose for RhaS/*P*_*rha*_ expression system, 0.25 mM 3-methylbenzoate for Xyls/*P*_*m*_ expression system (Supplementary Figure 4). The optimal condition of the T7 expression system was considered the same as that of the MmP1 expression system.

**FIGURE 6:**
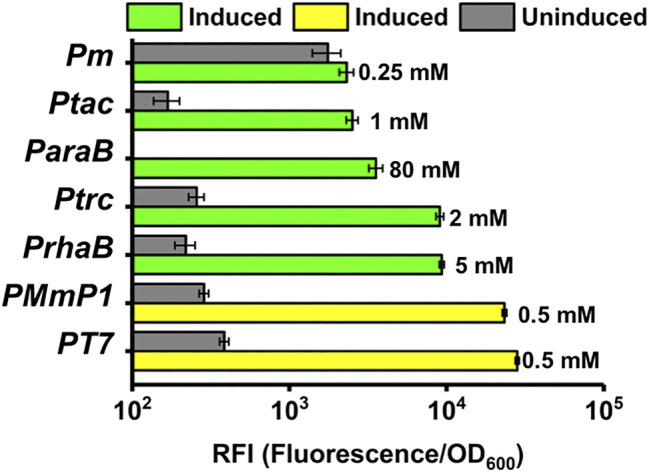
The RFI of the T7 and MmP1 expression system compared with the other inducible expression systems. The number to the right of each column represents the optimized inducer concentration.

The optimized induction conditions were then utilized to compare the expression level of different inducible systems in *P. putida* KT2440. As shown in Figure 6, a high expression level was achieved by the MmP1 expression system showing 2.5-fold, 2.6-fold, 6.6-fold, 9.3-fold, and 10.1-fold improvement compared to RhaS/*P*_*rha,*_ LacI^q^-*P*_*trc*_ AraC/*P*_*araB*_ Xyls/*P*_*m*_, and LacI^q^-*P*_*tac,*_ respectively. The expression level of the optimized T7 expression system was slightly higher than MmP1 by 1.2-fold. The expression level of RhaS/*P*_*rha*_ was followed by the LacI^q^-*P*_*trc*_ expression system and the AraC/*P*_*araB*_ expression system was lower than the LacI^q^-*P*_*trc*_ expression system, almost half of the RhaS/*P*_*rha*_ expression system. LacI^q^-*P*_*tac*_ and Xyls/*P*_*m*_ showed a weak expression level. All expression systems showed more than 10-fold improved sfGFP expression in induced conditions compared to uninduced conditions, except for Xyls/*P*_*m*_, specifically, the ratio of induced expression to uninduced expression was 73, 82, 43, 35, 123, and 15 for T7, Mmp1, RhaS/*P*_*rha*_, LacI^q^-*P*_*trc*_, AraC/*P*_*araB*_, and LacI^q^-*P*_*tac*_ expression system, respectively.

### Verification of the Effectiveness of the MmP1 Expression System by the Expression of NDHase

**FIGURE 7:**
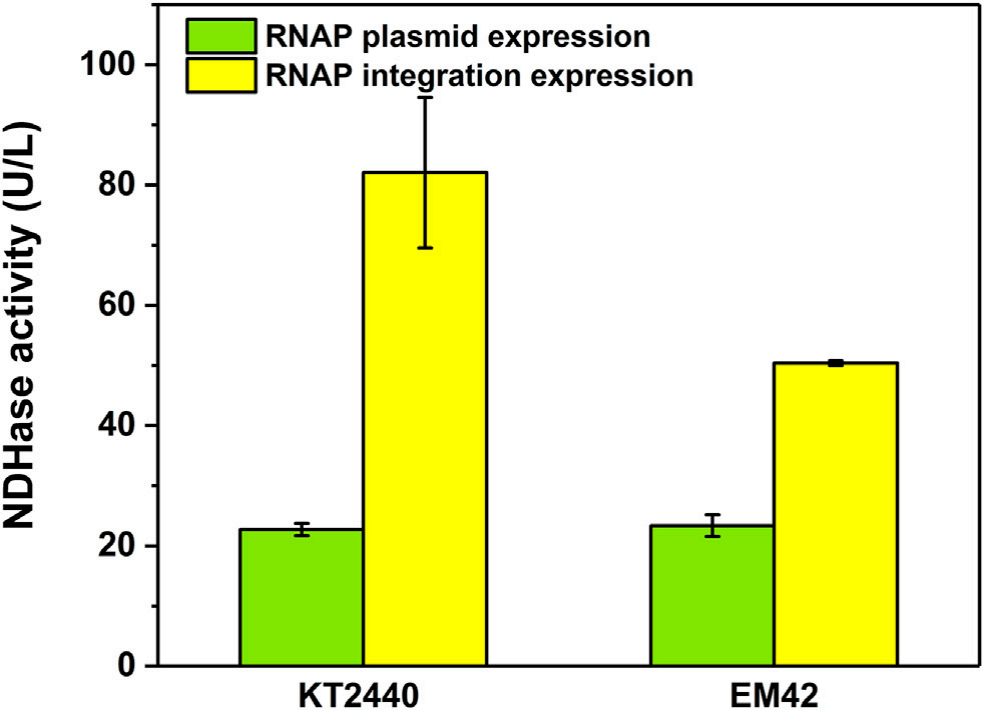
The activity of NDHase in the MmP1 expression system with the integration site of PP_5003 compared with the plasmid expression both in *P. putida* KT2440 and *P. putida* EM42.

The difficult-to-express NDHase from *C. testosteroni* JA1 was expressed using the MmP1 expression system both in KTCM and EMCM compared with KT2440 (Supplementary Figure 5). The activity of the NDHase using 3-cyanopyridine as substrate in the KTCM was 82.1 U/L, 3.6-fold higher than that in KT2440. The activity of the NDHase in the EMCM was 50.4 U/L, which is 2.2-fold higher than that in EM42 (Figure 7), exhibiting the excellent performance of the MmP1 expression system.

## Discussion

In previous research, the T7-like system MmP1 dramatically increased the GFP expression level in *P. entomophila* LAC31 (Zhao et al., 2017). Here, we would like to explore whether the T7-like system integrated into the genome of *P. putida* could also improve the expression level*.* We introduced the Mmp1 RNAP expression system both in the plasmid with the most effective origin of replication pRO1600/ColE1 (Damalas et al., 2020) and in the genome of *P. putida*. It was found that MmP1 RNAP was highly specific to the MmP1 promoter in *P. putida* KT2440 and the expression system was strictly regulated by IPTG (Supplementary Figure 2) with 82-fold induction. Previously, Zhao et al. found that one more Lac operator (*lac*O) introduced into the plasmid was necessary for the MmP1 expression system in *Halomonas* sp. TD01 (Zhao et al., 2017). However, *lacO* was not necessary for the MmP1 expression system in *P. putida* KT2440. Therefore, the MmP1 expression system was appropriate and convenient for heterologous expression in *P. putida* KT2440.

To optimize the MmP1 expression system in *P. putida,* we investigated the effect of insertion site and copy number on the heterologous protein expression. Three integration sites including *vdh, phaC1*, and *phaF* (locus tag is PP_3357 PP_5003 and PP_5007) were selected, which were reported to be nonessential genes and available insertion sites in *P. putida* (Gong et al., 2016). The RNAP cassette inserted in *phaC1* could significantly increase the expression of the sfGFP by 1.4-fold in KTCM, compared with the RNAP plasmid expression. Additionally, the RFI of sfGFP in KTCFM, KTFVM, and KTCFVM was lower than that in KT2440, indicating that the increased copy number of RNAP was not useful for the expression. Therefore, integrating one copy of the RNAP cassette in the site of *phaC1* in *P. putida* KT2440 was determined to be best for the improvement of expression level. And the MmP1 expression system in *P. putida* KT2440 was established, containing the host strain KTCM and plasmid pSEVA64-MmP1-sfGFP carrying its associated promoter P_MmP1_.

The induction conditions were then optimized by changing the induced point and the IPTG concentrations. It was finally determined that IPTG concentration of 0.5 mM and the induced point of 4 h after the inoculation provided the highly efficient heterologous expression in *P. putida* KT2440.

We then evaluated the versatility of the optimized MmP1 expression system in the genome-reduced strain *P. putida* EM42 showing much higher levels of recombinant protein production compared to *P. putida* KT2440 (Martínez-García et al., 2014). The MmP1 RNAP was inserted in the optimal integration site (*phaC1*) of the gene-reduced strain EM42 and successfully increased the expression of sfGFP by 2.1-fold in EMCM (Figure 4).

The optimal RNAP integration site (*phaC1*) was also used for introducing T7 RNAP in *P. putida* KT2440 and the expression level was enhanced as well, indicating the generality of the integration site for the T7 expression system. The MmP1 and T7 expression system were then compared with the existing expression systems in *P. putida* KT2440 (Calero et al., 2016; Martínez-García and de Lorenzo, 2017; Cook et al., 2018; Damalas et al., 2020). T7 and MmP1 expression systems both showed excellent performance than other cases and were nearly three times the LacI^q^-*P*_*trc*_ expression system, although the LacI^q^-*P*_*trc*_ expression system in *P. putida* KT2440 was improved almost 2-fold by switching *P*_*lacIq*_ with weaker constitutive promoters (Cook et al., 2018). This demonstrated that the MmP1 expression system constructed in this study was extremely effective.

The expression level of RhaS/*P*_*rha*_ was followed by the AraC/*P*_*araB*_ expression system, consistent with previous reports (Calero et al., 2016), but opposite to Damalas et al. (2020). The Xyls/*P*_*m*_ expression system seemed to be the weakest, which was similar to the previous reports (Damalas et al., 2020), but opposite to the findings of Calero et al. (2016). This could be due to the different origin of replication and the different ribosome-binding sites we used. In the previous study, T7 RNAP was integrated into the downstream of the *glmS* gene showing a lower expression level than RhaS/*P*_*rha*_ and AraC/*P*_*araB*_ expression system (Calero et al., 2016). In this study, we chose different insertion sites for T7 RNAP and ended up with more than 3-fold higher expression levels than other existing inducible expression systems (Figures 4, 5), suggesting that the insertion site was critical to effect of the RNAP.

To further confirm the utility of the established MmP1 expression system in *P. putida*, we performed the heterologous expression assay by using the difficult-to-express protein NDHase from *C. testosteroni* JA1. To our delight, the expression level of NDHase in both the engineered KTCM and EMCM were highly improved compared to the original strains. By using 3-cyanopyridine as substrate, the activities of whole-cell KTCM and EMCM were 82.1 and 50.4 U/L, which was almost 7- and 4-fold higher than that in the *P. entomophila* L48 (Yang et al., 2010).

Hence, we established the MmP1 and T7 expression systems in *P. putida* and provided a set of useful chassis (KTCM, KTCT, and EMCM) and corresponding plasmids (pSEVA64-MmP1-sfGFP and pSEVA64-DE-sfGFP) to express difficult-to-express proteins in synthetic biology. These expression systems also widened the application of T7-like RNA polymerase-based transcriptional regulation on the genome of *P. putida*. In addition, the strategy of optimizing the insertion sites discussed in the present study could potentially be used for enhancing the heterologous expression level for other non-model microbial strains.

## Data Availability

The raw data supporting the conclusions of this article will be made available by the authors, without undue reservation.
