# Supplementary material for: Construction of T7-Like Expression System in Pseudomonas putida KT2440 to Enhance the Heterologous Expression Level
Source: Front Chem. 2021 Jul 16;9:664967. doi: 10.3389/fchem.2021.664967 (PMC8322953; doi:10.3389/fchem.2021.664967)
Supplement: Supplementary file 1 [file DataSheet1.docx]

Supplementary Material

**Figure S1. Verification of the MmP1 integrated strains.**

Samples: lane M, 250bp DNA Marker.

KT2440 was for control.

KTVM: KTVM with MmP1 RNAP cassette integrated into *vdh* site of KT2440 harboring the plasmid containing sfGFP with MmP1 promoter;

KTFM: KTFM with MmP1 RNAP cassette integrated into *phaC1* site of KT2440 harboring the plasmid containing sfGFP with MmP1 promoter;

KTCM: KTCM with MmP1 RNAP cassette integrated into *phaF* site of KT2440 harboring the plasmid containing sfGFP with MmP1 promoter.

**Figure S2.** **GFP fluorescence was detected with or without IPTG in liquid medium.**

Left: cells under white light; Right: cells under blue light.

**Figure S3. MmP-1 expression system was strictly regulated by IPTG in *P. putida*.**





**Figure S4.** **Optimization of the induction condition for different expression system in *P. putida.*** A. Optimization of the induction point of MmP1 expression system in *P. putida*; B-G. Optimization of the inducer concentration of the inducible expression system in *P. putida*: B. Mmp1 expression system; C. LacI^q^/*P_tac_* expression system; D. LacI^q^/*P_trc_* expression system; E. AraC/*P_araB_* expression system; F. RhaS/*P_rha_* expression system; G. Xyls/*P_m_* expression system.

**Figure S5.** **Compared the expression of the ﻿nicotinate dehydrogenase in *P. putida* with pPM expression system through SDS-PAGE.**

M: protein marker; W: whole cell lysate; S: supernatant; P: ﻿precipitate. 1: KTCM: pSEVA64-JA; 2: KT2440: pSEVA64-JA; 3: EMCM: pSEVA64-JA. ndhL, ndhM and ndhS were ﻿three subunits of nicotinate dehydrogenase.

**Table S1. Strains and plasmids in this study.**

| **Strain** | **Description** | **Reference** |
| --- | --- | --- |
| **KT2440** | Wild type (mt-2 derivative, clearance of the TOL plasmid pWW0) | Nelson et al.(Nelson et al., 2002) |
| **EM42** | *P. putida* KT2440 derivative; Δ*prophage1* Δ*prophage4* Δ*prophage3* Δ*prophage2* Δ*Tn7* Δ*endA-1*Δ*endA-2* Δ*hsdRMS* Δ*flagellum* Δ*Tn4652* | (Martínez-García et al., 2014) |
| ***P. putida* KTVT** | *P. putida* KT2440 Δ*vdh* ::T7 RNAP cassette | This study |
| ***P. putida* KTVM** | *P. putida* KT2440 Δ*vdh* ::Mmp1 RNAP cassette | This study |
| ***P. putida* EMVT** | *P. putida* EM42 Δ*vdh* ::T7 RNAP cassette | This study |
| ***P. putida* EMVM** | *P. putida* EM42 Δ*vdh* ::Mmp1 RNAP cassette | This study |
| ***P. putida* KTCM** | *P. putida* KT2440 Δ*PhaC1* ::Mmp1RNAP cassette | This study |
| ***P. putida* EMCM** | *P. putida* EM42 Δ*PhaC1* ::Mmp1 RNAP cassette | This study |
| ***P. putida* KTFM** | *P. putida* KT2440 Δ*PhaF* ::Mmp1RNAP cassette | This study |
| ***P. putida* EMFM** | *P. putida* EM42 Δ*PhaF* ::Mmp1 RNAP cassette | This study |
| ***P. putida* KTCVM** | *P. putida* KT2440 Δ*PhaC1* ::Mmp1RNAP cassette; Δ*vdh* ::Mmp1 RNAP cassette | This study |
| ***P. putida* KTFVM** | *P. putida* KT2440 Δ*PhaF* :: Mmp1RNAP cassette; Δvdh ::Mmp1 RNAP cassette | This study |
| ***P. putida* KTCFM** | *P. putida* KT2440 Δ*PhaC1* ::Mmp1RNAP cassette; Δ*PhaF* :: Mmp1RNAP cassette | This study |
| ***P. putida* KTCFVM** | *P. putida* KT2440 Δ*PhaC1* ::Mmp1RNAP cassette; Δ*PhaF* :: Mmp1RNAP cassette; Δ*vdh* ::Mmp1 RNAP cassette | This study |
| **Plasmid** |  |  |
| **pMmP1-p321** | ﻿p321 derivates, with Mmp1 RNAP module and Mmp1 promoter module on it | Zhao, H. et al.(Zhao et al., 2017) |
| **pCAS-RK2K** | oriRK2, Kan^R^, Pcas-Cas9, ParaB-Red, PrhaB-sgRNA-pRO1600, SacB | Lab stock |
| **pSEVA-gRNAF** | oriPRO 1600/ColE1, Gm^R^, Pj23119-sgRNA-NicC | Lab stock |
| **pSEVA 64-vdh** | oriPRO 1600/ColE1, Gm^R^, N20, H1, H2 | This study |
| **pSEV 64-T7-vdh** | oriPRO 1600/ColE1, Gm^R,^, N20, H1, T7 RNAP, H2 | This study |
| **pSEVA 64-MmP1-vdh** | oriPRO 1600/ColE1, Gm^R^, N20, H1, MmP1 RNAP, H2 | This study |
| **pSEV 64- MmP1-5003** | oriPRO 1600/ColE1, Gm^R,^, N20, H1, MmP1 RNAP, H2 | This study |
| **pSEV 64- T7-5003** | oriPRO 1600/ColE1, Gm^R,^, N20, H1, T7 RNAP, H2 | This study |
| **pSEVA 64-MmP1-5007** | oriPRO 1600/ColE1, Gm^R^, N20, H1, MmP1 RNAP, H2 | This study |
| **pSEVA 64-DE-pRO** | oriPRO 1600/ColE1, Gm^R^, lacI, pTac promoter, T7 RNAP, T7 promoter, sfGFP | This study |
| **pSEVA 64-DE-sfGFP** | oriPRO 1600/ColE1, Gm^R^, T7 promoter, sfGFP | This study |
| **pSEVA 64-MmP1-pRO** | oriPRO 1600/ColE1, Gm^R^, lacI, pTac promoter, MmP1 RNAP, MmP1 promoter, sfGFP | This study |
| **pSEVA 64-MmP1-sfGFP** | oriPRO 1600/ColE1, Gm^R^, MmP1 promoter, sfGFP | This study |
| **pSEVA 64-JA** | oriPRO 1600/ColE1, Gm^R^, MmP1 promoter, NDHase | This study |
| **pSEVA 644-sfGFP** | oriPRO 1600/ColE1, Gm^R^, *lacI*^q^-P_trc_, sfGFP | This study |
| **pSEVA 644R-sfGFP** | oriPRO 1600/ColE1, Gm^R^, *lacI*^q^-P_tac_, sfGFP | This study |
| **pSEVA 648-sfGFP** | oriPRO 1600/ColE1, Gm^R^, *xylS/P_m_*, sfGFP | This study |
| **pSEVA 6410-sfGFP** | oriPRO 1600/ColE1, Gm^R^, *araC/P_araB_*, sfGFP | This study |
| **pSEVA 64-Rha-sfGFP** | oriPRO 1600/ColE1, Gm^R^, *rhaS/rhaR/P_rha_*, sfGFP | This study |

**Table S2. Primers used in this study.**

| **Primers name** | **Primer sequence (5’ → 3’)** |
| --- | --- |
| vdh-gF | TGGAACGCCCCGGTGATACGTTTTAGAGCTAGAAATAGCAAG |
| vdh-gR | TATCACCGGGGCGTTCCACGCTAGCATTATACCTAGG |
| vdh-1F | ATCCATTTGCGGCCGCTTTACCTTGGGCAGCTTGGTCGAG |
| vdh-ZR | ACAAGAGGAATGAGCCCGCGGCAGGCCCAGACCTCCGGCAAGATG |
| vdh-ZF | CTGGGCCTGCCGCGGGCTCATTCCTCTTGTTGTCGTTATAGAGAA |
| vdh-2R | TAGTTGCTCTAGAGCACCCTGAACCGGGAAATGGTCGACG |
| 5003-gF | GGGTTGGACAGGGTATTGGTGTTTTAGAGCTAGAAATAGC |
| 5003-gR | ACCAATACCCTGTCCAACCCGCTAGCATTATACCTAGGAC |
| 5003-U-F | CTCGGTACCCGGGGATCCCATCCTGCAGTTCGGCAAGATC |
| 5003-U-R | GCCACGGCGCTGTAACCTACGACGCTCCGTTGTCCTGAG |
| 5003-D-F | CGTCGTAGGTTACAGCGCCGTGGCGGCCTGCAC |
| 5003-D-R | GGAGTCCAAGACTAGTGCTGGCCATCATCCACAGCACC |
| 5003-mmp-R | GACTGGAAAGCGGGCAGTGACTACGACGCTCCGTTGTCCTGAGAC |
| 5003-mmp-F | CCGATTTTGCATTCGCTTAAGTTACAGCGCCGTGGCGGCCTGCAC |
| mmp-gRNA-5003-R | TTAAGCGAATGCAAAATCGGATTTCAGAATGTCTTGCAG |
| 5003-D-F | GTTACAGCGCCGTGGCGGCC |
| DE3-5003-R | GGCCGCCACGGCGCTGTAACTTACGCGAACGCGAAGTCCG |
| 5003-DE-R | GACTGGAAAGCGGGCAGTGACTACGACGCTCCGTTGTCCT |
| DE-5003-F | TCACTGCCCGCTTTCCAGTC |
| 5007-gF | GCCCCGCCAGCCAGATCTTGGTTTTAGAGCTAGAAATAG |
| 5007-gR | CAAGATCTGGCTGGCGGGGCGCTAGCATTATACCTAGGAC |
| 5007-D-F | GGTACCTGCTCTCCTTATGGTTTGTGCGCGACGCGCTC |
| 5007-D-R | CAGGAGTCCAAGACTAGTCCCTACCTTAATGCTCAAGC |
| 5007-U-F | GCTCGGTACCCGGGGATCCAGGATTACTGGCTGTTTCTGCAC |
| 5007-U-R | CCATAAGGAGAGCAGGTACCGCGTCGCCTTCTTCGCG |
| 5007-mmp-F | GAAGAAGGCGACGCGGTATCACTGCCCGCTTTCCAGTCGGGAAAC |
| 5007-mmp-U-R | CTGGAAAGCGGGCAGTGATACCGCGTCGCCTTCTTCGCG |
| 5007-D-mmp-F | GCGGCCTTTTTTCGTTTTGGTCCCCTGCTCTCCTTATGGTTTGTGCG |
| 5007-mmp-R | GGACCAAAACGAAAAAAGGCCGC |
| sfGFP-R | TCATTTGTACAGTTCATCCATACCATGCG |
| sfGFP-F | ATGCGTAAAGGCGAAGAGCTG |
| Rha-sfGFP-R | AGCTCTTCGCCTTTACGCATGGTATATTCCTCCTATCGCC |
| Rha-sfGFP-F | GGATGAACTGTACAAATGAACTAGTCTTGGACTCCTGTTG |
| xyls-sfGFP-F | GATGAACTGTACAAATGACTTGGACTCCTGTTGATAGATCC |
| tac-F | TCACTGCCCGCTTTCCAGTC |
| tac-R | CCTCCTATCGCCCTTAAGCTCCTTTGTGAAATTGTTATCC |
| tac-sfGFP-F | GGCGATAGGAGGAATATACCATGCGTAAAGGCGAAGAGCT |
| tac-sfGFP-R | GACTGGAAAGCGGGCAGTGATTAATTAAAGGCATCAAATA |
| ara-F | TTAATTAATTATGACAACTTGACGGCTACATCATTC |
| ara-R | CCTCCTATCGCCCTTAAGATGGAGAAACAGTAGAGAGTTG |
| ara-sfGFP-F | CTTAAGGGCGATAGGAGGAATATACCATGCGTAAAGGCGA |
| ara-sfGFP-R | CAAGTTGTCATAATTAATTAAAGGCATCAAATAAAACG |
| trc-F | GATGCCTTTAATTAAGACACCATCGAATGGTGCAAAAC |
| trc-R | CTTAAGCATGGTCTGTTTCCTGTGTGAAATTGTTATCCGC |
| trc-sfGFP-R | CGATGGTGTCTTAATTAAAGGCATCAAATAAAACGAAAGG |
| trc-sfGFP-F | GGAAACAGACCATGCTTAAGGGCGATAGGAGGAATATACC |
| mmp-JF | CTGACTGTTCCATTGATGCCCATAA |
| mmp-JR | CCTATTTTGCCTGTCCTTATGCGATTC |
| 5003-JF | GCTCGAAGAAGTGTCGTTCAGTGC |
| 5003-JR | CAACGGGTCGTCGTCGCCAG |
| 5007-JF | GGCTGGACGCAGAGGATTACTG |
| 5007-JR | GAGCGATGTGATGTGAGGGTGG |
| DE-JF | GATAGCGAGATTGATGCACACAAACAG |
| JA-JF | CTGAACGGCCAGGAAGTGGAC |
| JA-JR | CTTGTTATCGCAATAGTTGGCGAAGTAATC |
| cas-F1-1 | GGGTGAGCTGCATGCTATTTTGAG |
| cas-R1-1 | CTCGCGAATTTTTCTGGCCCTTTTGA |
| PS5 | CCCTGCTTCGGGGTCATT |
| PS7 | AGATGCCGCTGGATCTGG |
| PS1 | AGGGCGGCGGATTTGTCC |
| PS2 | GCGGCAACCGAGCGTTC |

**Table S3. Information on the chromosomal insertion sites of different RNAP cassette.**

| gene | Length(bp) | Insertion site and length(bp) |
| --- | --- | --- |
| T7 RNAP cassette | 4097 | PP_3357 (*vdh*) /1449 |
| Mmp1 RNAP cassette | 4097 | PP_3357 (*vdh*) / 1449 |
| T7 RNAP cassette | 4097 | PP_5003 (*phaC1*) /1680 |
| Mmp1 RNAP cassette | 4097 | PP_5003 (*phaC1*) /1680 |
| Mmp1 RNAP cassette | 4097 | PP_5007 (*phaF*) /786 |
